# Ethical considerations in the conduct of research on therapies for the prevention and treatment of Ebola virus disease in developing countries

**DOI:** 10.11694/pamj.supp.2015.22.1.6176

**Published:** 2015-10-10

**Authors:** Morenike Oluwatoyin Folayan, Bridget Gabrielle Haire

**Affiliations:** 1 Obafemi Awolowo University, Ile-Ife, Osun State, Nigeria A234; 2 Bridget Gabrielle Haire, Kirby Institute for Infection and Immunity in Society, Wallace Wurth Building, UNSW Australia, Sydney, NSW 2052, Australia

**Keywords:** Ethics, research, Ebola, developing countries

## Abstract

The devastating toll of the Ebola epidemic in West Africa necessitates considerations of new approaches to research into new prevention technologies and treatments for Ebola Virus Disease (EVD). Research must be planned and delivered in consultation with civil society from the epicentre to prevent mistrust and misunderstanding. Ethical considerations include development of local research and regulatory capacity; negotiating the standard of prevention packages for research participants, including healthcare workers; and strengthening health systems in developing countries to ensure effective response to future EVD outbreaks in the region. Also, strategic consultation with local communities is an ethical imperative for EVD research, particularly where there is potential for differential access to prevention and care packages between trial staff and local hospital staff.

## Essay

The increasing incidence of cross-border infection exemplified by case detection in Nigeria, Mali, Senegal, England, United States and Spain, further necessitates consideration of new approaches to the evaluation and distribution of new treatment and prevention technologies. Despite possible remission of EVD symptoms following early detection and aggressive supportive care, mortality reported in the current epidemic has been estimated to be 73% [1] with mortality rates from previous outbreaks ranging between 25% and 90%. The current epidemic has caused the loss of more than 8,439 lives [2].

The high case fatality may be connected to poor medical management of patients with EVD [3]. Provision of supportive therapy through replacement of body fluids, electrolytes and proteins remain the mainstay of therapy for EVD in the absence of any curative of preventive therapy [4]. Unfortunately, the magnitude of the infection has led to compromised quality of care for patients, with the restrictive personal protective equipment (PPE) required for healthcare workers constraining some aspects of care, thus further limiting supportive therapy and increasing case fatality [3].

Development of EVD treatment drugs and vaccines is very important, but glaringly, development of local capacity to provide care and support for patients with EVD is equally essential. Accordingly we will also address issues of local capacity in this paper, which will discuss and identify significant ethical considerations when planning the design and implementation of clinical trials for EVD treatment drugs and vaccines.

### Design of clinical trials for interventions intended to prevent or treat EVD

Phase I trials for anti-EVD vaccines and therapeutics should happen in developed countries as is the norm with other drug and vaccine development process. This is because of the need to ensure optimum standards of clinical care and patients’ monitoring during phase I trials while establishing baseline safety data. Phase II trials however may be conducted in developing countries with EVD outbreaks. This should not preclude efforts to build local capacity in Africa to conduct phase I clinical trials. Kanapathipilla et al [5] proposed that phase II trials can be split into IIa trials conducted in people at low risk of the target infection, and phase IIb, conducted in those at higher risk of the target infection. This would help to establish the optimal schedule for efficacy trials and build a hypothesis regarding efficacy that would dictate sample size and statistical power in later trials. This suggests that phase IIa trials could be conducted outside the Ebola outbreak zone in Africa and phase IIb trials conducted within it, in populations at high risk. These trials can be conducted in parallel [5].

For EVD vaccine research, Kanapathipilla et al [5] proposed the implementation of a “stepped wedge” cluster trial design with randomisation occurring at community level rather than the individual. This approach would enable communities severely affected by Ebola to be randomised, introducing the vaccine one community at a time, with the goal being that all communities would get access in a staged process.

Unfortunately, countries affected by EVD in West, East and Central Africa have grossly inadequate health infrastructure and do not have a large pool of personnel with competency to conduct clinical trials [5]. In addition, funding of research of tropical and neglected diseases like EVD has been poor and has been auspiced mainly by external donors rather than by governments of affected countries [6]. Further, the research regulatory agencies in these countries have low capacity to address ethical issues that arise and provide regulatory oversight for the trials [7]. In view of these limitations, Rid and Emmanuel argued that the global community had an ethical obligation to support the development of therapies for the management and control of EVD in the affected region [8]. Current support has been largely in the form of funding EVD research. There is however concern that the investment in EVD research may draw away from the current limited funding of research into other tropical neglected diseases [3]. Once infrastructures and systems are in place, researchers can draw on local funding, however limited in scope and size, to conduct research to address local needs. This way, the EVD research enterprise can leave an enduring positive legacy in the EVD region long after the current EVD outbreak has been brought under control.

### Standard of prevention and local realities

While most if not all of the EVD clinical trials protocols for treatment therapies and vaccine are developed primarily by researchers in the developed countries, this research will be conducted in collaboration with partners in the local settings. For EVD treatment research, health care workers will be actively engaged with drug administration and data collection. Also, healthcare workers are to be targeted as study participants for EVD vaccine research due to their heightened risk for contracting EVD [9]. It is therefore important to give due consideration to the standard of EVD prevention packages for healthcare workers who will be engaged in EVD therapeutic and vaccine research as care providers and as study participants.

For healthcare workers as care providers, the standard of prevention package should include providing effective personal protective equipment (PPE) and implementing evidence-based standardised protocols regarding working conditions (time spent within suits, procedures for robing and disrobing among other things). There is evidence that suggests a need to improve the quality of PPE [10] as the current standard is uncomfortable to work in, limits work efficiency [3] and can be permeable [10]. This same consideration needs to be given to provision of PPE for healthcare workers who participate as volunteers in EVD vaccine clinical trials, as it is imperative that every effort must be made to prevent undue exposure of research participants to risk of EVD infection.

In addition, due consideration should be given to the PPE requirements of those who wear prescription glasses and the use of fluid resistant particulate respirators when carrying out procedures that could cause aerolisation of infectious particles [11]. While Ebola is not generally airborne, in the context of some health care practices like intubation, the likelihood of inhaling particles may be increased. Although we do not make a blanket recommendation for respirators for all healthcare workers regardless of the tasks they are undertaking, the guidelines published by the World Health Organisation [12] and Centre for Disease Control (CDC) [13] now recommend their use in some contexts. The feasibility of using respirators for some procedures will need to be negotiated with healthcare workers. Efforts also need to be invested in the development of evidence-based guidelines for EVD management.

Furthermore, participants and researchers will need to be carefully prepared to deal with fever that could arise as a complication of EVD vaccine research. Fever is a common vaccination side effect and, not unexpectedly, has been seen in a small number of participants in the human safety trials of one of the candidate Ebola vaccines [13]. Fever is also primary screening tool for detection of people with suspected EVD. This side effect of EVD vaccine will have to be managed very carefully to prevent social harms as there is the potential that healthcare workers who are vaccinated for EVD could also be identified as possibly having acquired EVD. The corollary is that study participants could be placed in quarantine. Unfortunately, the state of quarantine facilities in EVD affected countries is so poor that people have stayed home with EVD rather than turn up at the quarantine centres. If study participants with fever have to stay in these quarantine centres along with others identified as having been exposed to EVD, this would heighten their risk of being infected to an unacceptable level. Due consideration therefore needs to be given to the management of fever detected during EVD vaccination and the implications for quarantining study participants who come down with fever.

### Standard of care and local realities

As healthcare workers face increased risk of Ebola exposure and infection in their daily lives, it is also likely that some healthcare workers who volunteer for the EVD vaccine trials will acquire EVD. This raises the issues of the standard of care for EVD trial participants who become infected. The long-standing debate on what constitutes appropriate standard of care in resource constrained settings [14] has once again arisen with respect to EVD research participants’ management. For example, most of the EVD vaccine trials are proposed by partners in developed countries and would be implemented largely by partners in affected countries. However, how would EVD care be handled if and when a healthcare worker from a developing country and a healthcare worker from a developed country contracts EVD during the trial? Is there a justification for differential access to medical care in trial situations as is the case with clinical care which Kass et al [15] had justified? While it is hard to justify such a differential standard of care between healthcare workers on a clinical trial based on their nationality, providing higher standard of care to trial participants than for those in the immediate communities who acquire EVD but are not on the trial is also inequitable. Differential access to standards of medical care by routine clinical patients and trial participants has given rise to community tensions in the past [16, 17].

The standard of care package for trial participants will need to be carefully negotiated to optimise care not just for participants but also for their local communities. In the context of HIV prevention trials, negotiation of standard of care packages has also been proposed, especially in situations where the standard of care between developing and developed countries differ significantly, and where the institution of international gold standard of practice may involve differing standard of care between research participants and other community members [18]. Discussing standard of care packages for EVD research and making clear the reasons for any differences between categories of individuals would help prevent mistrust.

### Capacity building for EVD research

A further ethical imperative for trial conduct in countries with EVD epidemics hosting EVD research is the need to ensure technology transfers. It is very clear that the development of EVD therapeutics and vaccines can no longer be delayed. The corollary of this is that research teams need to think about how to develop not only the infrastructure but also the human and administrative capacities in proposed research sites to facilitate the conduct of the required phase II and III trials. There is a need for structured supportive systems that promote local capacity building for research staff in Ebola-affected countries in ways that enable them to design and implement future research studies that can address local EVD containment. Unfortunately, the lion's share of the funding for research on Ebola is currently spent in developed countries outside Africa, with very little focus on building the capacity of researchers in affected region been able to lead Ebola research initiatives [6]. This paradigm would need to change in due recognition of the collective global responsibility to build local capacity in Africa to address their health needs.

### Sharing decision making

The ethical complexities of conducting clinical trials during epidemics of such magnitude as the current West Africa outbreak make it imperative that decisions about compassionate access for therapy and clinical trial design for EVD therapeutics and vaccines are made in consultation with affected communities, including non-medical members of civil society. Building shared understandings of the epidemic and research strategies to help affected people and communities are critical to the success of research and access programs, making the involvement of civil society in EVD research planning and implementation crucial.

In an epidemic setting where the prospect for compassionate access to new interventions is possible, decisions about the conduct of clinical trials of such interventions has to be weighed against compassionate access for those infected or at high risk of infection. Compassionate access is important for humanitarian reasons, and it may also assist with gaining compliance with public health measures. Access to an experimental vaccine should however be based on defined clinical criteria that allow for systematic data collection, without preferential treatment for people with particular roles in society, in order to avoid entrenching social inequalities [19].

Compassionate access to experimental therapeutics and vaccines may however deplete the supply of experimental interventions and complicate evaluative processes, as patient deaths or recoveries may be wrongly attributed to the experimental substances. This and other possible challenges with evaluation of the efficacy of potential EVD therapeutics and vaccines should not preclude access of citizens in a country where EVD therapeutics and vaccines clinical trials are been conducted to compassionate therapy.

In a health crisis like that been currently experienced with EVD in West Africa, civil society consultation may sound like an unaffordable luxury. The decisions that need to be made over the following months about how to distribute and test experimental therapeutics and vaccines in West Africa will necessarily involve complex choices based on values, however.

Without meaningful input from civil society, and strong support from the media for disseminating accurate and unbiased information, there is the risk that EVD therapeutics and vaccine research and development could exacerbate social inequalities and breed social unrest. Perceptions about social injustice could arise from differential access of trial participants and trial support staff to prevention and care packages that dichotomise the populace.

Decisions therefore need to be made with communities on research design and implementation plans including participant recruitment process, whether or not some communities are too vulnerable to participate, and how to ensure communication of key concepts to community members who participate in clinical research. The decision-making process should promote collaboration between researchers and community members as invested stakeholders in the management and control of the EVD epidemic. Dialogue between researchers and communities should therefore be bidirectional and continuous up till the point of research result dissemination. Community-research partnerships should be the ultimate goal of the community engagement process for EVD therapeutics and vaccine clinical research programmes. The Good Participatory Practice Guidelines [20] developed to guide community-research interactions for HIV prevention research can serve as a good reference document for developing community engagement programme for EVD clinical research.

## Conclusion

Development of effective EVD treatment drugs and vaccines must be a global priority. Prevention however cannot be the only strategy for combatting this disease. Pictures and reports from countries affected by the EVD have shocked the world, with men, women and children suffering and dying in unspeakable conditions that attest to the parlous state of health infrastructure in areas stricken by the disease. The social and economic effects of the epidemic will further delay the development of infrastructure that is urgently needed to provide decent care to people in these countries. Strengthening the health systems of countries with low GDP needs to be given global priority while effective EVD therapies are been researched and developed. The next epidemic may hit another region in Africa outside the current know zones of the epidemic. The world cannot afford a repeat of the current EVD epidemic we are witnessing.
